# The golden hour of acute ischemic stroke

**DOI:** 10.1186/s13049-017-0398-5

**Published:** 2017-05-22

**Authors:** Rajiv Advani, Halvor Naess, Martin W. Kurz

**Affiliations:** 1 Department of Neurology, Stavanger University Hospital, Postboks 8100, Stavanger, 4068 Norway; 2 Neuroscience Research Group, Stavanger University Hospital, Stavanger, Norway; 3 Department of Neurology, Haukeland University Hospital, Bergen, Norway; 4 Institute of Clinical Medicine, University of Bergen, Bergen, Norway

## Abstract

**Background:**

Acute Ischemic Stroke (AIS) treatment has been revolutionised in the last two decades with the increasing use of Intravenous Thrombolysis (IVT) and with the advent of Endovascular therapy (EVT). AIS treatment and outcome are time dependant and time saving measures are being implemented at every step of the treatment chain. These changes have resulted in lower treatment times in-hospital, but it is unclear if this translates into more patients being treated within 60 min of symptom onset – the Golden Hour. The clinical outcome of IVT therapy in this patient group was our secondary outcome.

**Methods:**

From 2009 onwards, systematic changes were made to the AIS treatment chain leading to a dramatic decrease in Door-to-Needle (DTN) time. Analyses were performed on the number of these treatments year on year and their clinical outcomes within the Golden Hour at Stavanger University Hospital (SUS).

**Results:**

Six-hundred and thirteen patients were included; seventy-three were treated within the Golden Hour. The percentage of total IVT treatments occurring in the Golden Hour rose from 2.2% in 2009 to 14.5% in 2015 (*p* = 0.006) with a high of 18.3% in 2012 (*p* < 0.001). All of these patients had a Median NIHSS of 0 at discharge, irrespective of age and pre-existing comorbidity. There was no incidence of any ICH and in-hospital mortality was only 2.7% in this group.

**Discussion:**

The time from AIS symptom onset to treatment is filled with delays. Despite the inherence of some delays,significant efforts on the part of the pre- and in- hospital treatment chain have made IVT therapy within 60 min a possibility. The allocation and use of resources in the setting of rapid AIS treatment is warrantedand yields unprecedented results.

**Conclusions:**

Our study shows that improved treatment routines led to an increase in the number of patients treated within the Golden Hour. Treatment in the Golden Hour leads to excellent outcomes in all patients, irrespective of age and pre-existing comorbidity.

## Background

The last two decades have seen a revolution in the treatment of acute ischemic stroke (AIS), initially with the use of intravenous thrombolysis (IVT) [1–3] and more recently with the advent of endovascular thrombectomy (EVT) [4–6]. An increasing number of patients have had access to therapy over recent years due to improvements in pre- and intra- hospital systems [7, 8]. However, both these therapeutic modalities are time dependant and analyses have shown that better outcomes can be expected when treatment is administered sooner rather than later within the therapeutic window [9–11].

Recent analyses into outcomes of treatment have shown that treatment within 60 min of symptom onset produces excellent outcomes with significantly lower rates of morbidity and mortality in younger patients [12]. The authors suggest that the results are so encouraging that pre-hospital IVT should be the way forward in AIS treatment. This remarkable 60 min window from the onset of symptoms has been increasingly known as The Golden Hour.

However, IVT therapy in the pre-hospital setting is fraught with challenges, a recently published editorial highlights these challenges and errs on the side of caution [13].

At our treatment centre, Stavanger University Hospital (SUS), we have worked systematically since 2009 to reduce the time used in-hospital to treat patients with AIS. The aim of these changes was to reduce the Door-to-Needle (DTN) time. The successfully implemented organisational changes, described in detail in earlier publications [7, 14], reduced the median DTN time to under 30 min. Our results also showed that transport times to SUS remained unchanged in the period of our study.

Reducing DTN time shows increased efficacy on the part of the AIS treatment chain, but does this translate into a reduction in Onset-to-Needle (OTN) time – do we treat more patients within The Golden Hour?

Our primary aim was to assess if the falling DTN time was associated with an increase in the number of patients with an OTN time of 60 min or less. Our secondary aims were to assess the clinical outcomes these patients had to see if early treatment correlated with good outcome in our patient population.

## Methods

### Patient population/catchment area

SUS serves a population of almost 350 000, receiving all AIS patients in its catchment area. Approximately 200 000 inhabitants live in and around the city of Stavanger; the remainder live in more rural areas of Rogaland County.

All IVT treatments at SUS during the 2009 to 2015 (*n* = 613) are included, irrespective of age, sex, other comorbidities and stroke severity on admission. According to treatment guidelines IVT was administered within 4.5 h of AIS symptom onset. These numbers are inclusive of any stroke mimic diagnoses (both medical and functional) that presented as AIS on admission.

### Stroke database

All patients treated with IVT were retrospectively compiled into a database until 2012, when prospective inclusion began in our stroke database. This database included details from medical records including AIS risk factors, National Institutes of Health Stroke Scale (NIHSS) scored on admission, after IVT therapy and at discharge; treatment complications and in-hospital mortality were also noted. All the applicable times including; time of stroke onset, when the EMS were contacted, the time of admission to the hospital and time of IVT administration were taken from a combination of the pre-hospital records (AMIS – Acute Medical Information System) and hospital medical records. The relevant time durations including DTN and OTN time were then calculated.

All these data were compiled into a single database.

### Clinical follow-up

Patients with suspected AIS were admitted directly to the ER, where clinical examination to assess NIHSS was performed followed by a head CT (Computed Tomography). Once contraindications had been excluded using the head CT, IVT treatment was administered. Following the IVT bolus, a CT angiography and CT perfusion scan were also performed to determine the presence of a Large Vessel Occlusion (LVO). Patients with a LVO were transferred to the EVT lab and excluded from our analyses. After the administration of IVT, patients were admitted to a neuro-intensive unit for further clinical follow up. NIHSS scoring was performed two hours after IVT administration to assess the therapeutic effect and again at twenty-four hours. A follow-up head CT was performed between 12 and 24 h after IVT administration to determine the presence of any intracerebral hemorrhage (ICH). In patients with a positive clinical course no further imaging was performed and a final NIHSS assessment was performed on discharge. In those worsening or not showing clinical improvement, cerebral imaging was repeated along with NIHSS assessment at regular intervals. Stroke physicians were in-charge of patient follow up at the ward and the other relevant medical specialties were consulted in conjunction with any complications, i.e. myocardial infarction, infections, etc.

### Data analysis

The afore mentioned database was used to calculate certain key variables such as the DTN time, OTN time, NIHSS on admission and discharge. Using these variables we could ascertain the number of patients treated within the Golden Hour (OTN ≤ 60 min) year on year from 2009 up to and including 2015. We could then calculate the percentage of all IVT treatments occurring within 60 min of onset year by year. Using the NIHSS on admission and discharge we could ascertain whether patients treated within the Golden Hour improved after therapy and if they had neurological deficits if any. The diagnosis at discharge for each patient was recorded in the database so that we could keep track of stroke mimics. Any ICH and or in-hospital mortality was also recorded.

### Statistics

Using the Stroke research database, the data for the above mentioned end points was collected and analyzed. Statistical analyses were conducted to determine any changes in the number of EMS admissions as well as the number of treatments with IVT. Statistical analysis was conducted using IBM SPSS version 22. *P* values were determined using one-way analysis of variance (ANOVA) and Pearson’s Chi squared test as appropriate. *P* values less than 0.05 were considered significant.

## Results

**Fig. 1:**
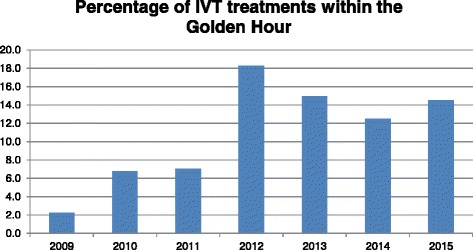
The percentage of all IVT treatments occurring within the Golden Hour from 2009 – 2015

Figure 1 shows the percentage of all IVT treatments with an OTN time of less than or equal to 60 min – i.e. within the Golden Hour. There is a rise in the percentage of patients treated within the Golden Hour from 2.2% in 2009 to 6.8% in 2010 and then 7.1% in 2011. In 2012 there is a dramatic increase in the percentage treated within 60 min of onset – 18.2% of all IVT treatments (*p* < 0.005). This number then falls to 14.9% in 2013 and to 12.5% in 2014 before rising to 14.5% in 2015 (*p* = 0.006).

Table 1 shows patients treated with IVT divided into the respective therapeutic windows. The first group comprises those treated within the Golden Hour (*n* = 73), the second group are those treated from 61 min up to and including 180 min (*n* = 414) and the final group are those receiving treatment from 181 min up to and including 270 min (*n* = 126) after symptom onset. The median age (range) of patients in the three groups was 71 (38–91), 72 (41–93) and 75 (42–94) respectively. Forty-one percent of the Golden Hour group suffered from hypertension, compared to Forty-six percent in the group treated between 61 and 180 min of onset and Fifty-seven percent amongst those treated between 180 and 270 min of symptom onset. There were no statistically significant differences between the groups. In the same groups the percentage of those currently smoking was 27%, 21% and 15% respectively, again no statistically significant difference was observed. In the same groups the percentages of those with a previously diagnosed atrial fibrillation was 15%, 11% and 19% respectively – these differences weren’t statistically different. In the Golden Hour group only 4% were diabetics, significantly lower than the 12% of diabetics in the group treated between 61 and 180 min (*p* < 0.005) and the 19% of those treated in the final therapeutic window (*p* < 0.005).

**Table 1:** The patient characteristics by therapeutic window

	Golden hour	61–180 min	181–270 min
Number of Patients	73	414	126
Median Age	71	72	75
Hypertension (%)	30 (41.1)	194 (46.9)	72 (57.1)
Currently Smoking (%)	20 (27.4)	90 (21.7)	19 (15.1)
Atrial Fibrillation (%)	11 (15.1)	49 (11.8)	25 (19.8)
Diabetes (%)	3 (4.1)	52 (12.6)	25 (19.8)

Table 2 shows the Clinical Course of the patients treated with IVT in the respective therapeutic windows. The median NIHSS (range) on admission in the six groups was quite similar 7.0 (2–13), 8.0 (2–13), 6.5 (2–14), 7.0 (2–14), 7.5 (2–14) and 7.0 (2–14) – no statistically significant difference was observed. The median NIHSS (range) at discharge in both Golden Hour groups is 0 (0–6), lower than 4 (1–11) and 5 (2–14) in the two subgroups treated between 61 and 180 min after onset (*p* < 0.005). The median NIHSS at discharge for those treated in the final therapeutic window was 7 in both subgroups, significantly higher than those in the Golden Hour group (*p* < 0.005). The percentage ICH was 0.0% in both age subgroups for those treated within the Golden Hour. The percentage ICH rose to 0.8% (*p* = 0.76) and 3.9% (*p* = 0.44) for those treated between 61 and 180 min. The percentage ICH for those aged ≤79 in the final therapeutic window was 8.0% (*p* = 0.02) and 19.4% (*p* < 0.005) for those aged 80 and over.

**Table 2:** The clinical course of IVT treatment in the different therapeutic windows including age sub-groups

	Golden hour	Golden hour	61–180 min	61–180 min	181–270 min	181–270 min
Age Group	≤79	>80	≤79	>80	≤79	>80
Number of Patients	46	27	261	153	62	67
Median NIHSS on Admission	7	8	6.5	7	7.5	7
Median NIHSS at Discharge	0	0	4	5	7	7
ICH (%)	0 (0.0)	0 (0.0)	2 (0.8)	6 (3.9)	5 (8.0)	13 (19.4)
In-hospital Mortality (%)	0 (0.0)	2 (7.4)	4 (1.5)	10 (6.5)	10 (16.1)	15 (22.3)

The in-hospital mortality is 0.0% for those aged ≤79, treated within the Golden Hour and 7.1% for those aged 80 and over. The in-hospital mortality for those aged ≤79 treated in the second therapeutic window is 1.5% and 6.5% for those 80 and older – no statistically significant difference is observed. In the final therapeutic window, the in-hospital mortality for those aged ≤79 is 16.1% (*p* < 0.005) and 22.3% (*p* < 0.005) for those 80 and older.

Twenty-five (4.1%) of our cohort left the hospital with a Stroke Mimic diagnosis; results not shown.

## Discussion

Our study showed a significant increase in the number of patients being treated within the Golden Hour as a result of the decreasing DTN – an increase from 2.2% to 14.5% (*p* = 0.006). Larger works have shown that less than 29% of AIS patients actually arrive at the Emergency Room (ER) within 60 min of onset and of those, only 18% have a DTN time of 60 min or less [15]. The benefits of getting to the hospital quickly are often nullified due to the stresses of the ER [16] thus very few patients are actually treated within 60 min of symptom onset. The fact that our center is consistently treating every eighth patient (18.2%, 14.8%, 12.5% and 14.5%) within the Golden Hour is quite remarkable.

The benefits of rapid treatment are clear in both age groups, with a median NIHSS of 0 at discharge, significantly better outcomes were achieved in the Golden Hour than in the later therapeutic windows. There was also no incidence of any ICH for octogenarians and nonagenarians treated with IVT within the Golden Hour. The fact that those aged over 80 achieved such results is of great significance for clinical practice as these patients usually comprise the majority of stroke deaths [17, 18]. They are usually also excluded from clinical trials and analyses making the decision to treat in the acute phase even more challenging [19].

The in-hospital mortality amongst those > 80 years old treated in the Golden Hour was also very low (7.4%). This is noteworthy due to the fact any hospital admission in octogenarians and nonagenarians is associated with significant in-hospital mortality (20–40%) [20, 21]. These two cases of in-hospital mortality were both nonagenarians having significant pre-existing comorbidity. Nevertheless, 97.3% of the octogenarians and nonagenarians had excellent outcomes of IVT therapy within 60 min of symptom onset without any incidence of ICH. With the expected exponential rise in the population of octogenarians and nonagenarians these results provide the firm basis for early treatment in these patients.

The work by Ebinger et al on Golden Hour IVT therapy excludes patients with a stroke mimic diagnosis [12] whereas our work has included these patients. Stroke mimics make up a very real part of patients in the ER. The incidence of mimics in our cohort (4.1%) is lower than in other studies [22], and could be due to the fact that mimics tend to complicate the decision to treat and are therefore associated with increased treatment times [23]. Nevertheless, the fact that this patient group can be treated without any ICH and or poor outcome is of clinical significance in the hyper acute setting. The finding that IVT treatment in stroke mimics is safe is also supported by other works [23, 24].

The retrospective nature of data collection in the earlier years (2009–2011) subjects our study to certain unavoidable weaknesses; however, the majority of patients were prospectively included. The merits of our study include the number of patients included and the number of years of our study. The homogeny of the patient population is also a strength of our study as it excludes any variation in outcomes due to race/ethnicity. The continued and ongoing work at our treatment center toward reducing OTN and DTN times could be a source of bias; the EMS crews and ER staff working more quickly to ensure shorter treatment times for patients with AIS. The bias is somewhat inevitable, but is an important byproduct of our focused work with the members of the treatment chain. We feel that ongoing efforts with the staff involved in the treatment of AIS patients is fundamental to providing rapid and efficacious treatment in the acute setting. The acute setting in the ER can provide unforeseen challenges and stresses, having every member of the team primed toward the goal of rapid treatment is inevitably advantageous.

The excellent outcomes associated with IVT treatment in the Golden Hour in all age groups including stroke mimics suggests that all efforts should be made to ensure treatment within this opportune therapeutic window. These results also reinforce that any efforts made, both pre- and in- hospital, are worthwhile investments in securing the best outcomes for the patient population. The routines surrounding rapid IVT therapy should be extended to elderly patients as they benefit equally from early treatment.

## Conclusions

Acute Ischemic Stroke, it’s treatment and thus it’s clinical outcomes are time dependent. Significant efforts are being made both pre- and in- hospital to provide treatment as quickly as possible. Our results show that treating patients within one hour of symptom onset leads to excellent outcomes, without any incidence of iatrogenic bleeds. These excellent outcomes are also seen in octogenarians, suggesting that age alone should not contradict rapid treatment. The protocols put in place to ensure rapid treatment yield excellent results and should be extended to all patients, irrespective of age.
